# Mendelian randomization analysis reveals causal roles of inflammatory cytokines in thyroid cancer pathogenesis

**DOI:** 10.1007/s12672-025-03533-9

**Published:** 2025-09-02

**Authors:** Bo Liu, Tingting Zhang, Jihua Han, Wen Bi, Chunlei Nie, Jiewu Zhang

**Affiliations:** 1 Department of Head and Neck Surgery, Harbin Medical University Cancer Hospital, No.150 Haping Road, Nangang District, Harbin, 150081 China; 2 Psychology and Health Management Centre, Harbin Medical University, No.157, Baojian Road, Nangang District, Harbin, 150076 China

**Keywords:** Thyroid cancer, Mendelian randomisation, Inflammatory cytokines, Causal relationship

## Abstract

Thyroid cancer is a common endocrine malignancy associated with various inflammatory factors. This research aimed to explore the causal relationships between 41 inflammatory factors and the risk of thyroid cancer using Mendelian randomisation (MR) analysis. MR analysis was performed using genetic data from two publicly available European genome-wide association studies (GWAS). Instrumental variables were selected based on single-nucleotide polymorphisms significantly associated with cytokine levels. Causal relationships were assessed using the inverse variance weighted method, with sensitivity analyses to evaluate heterogeneity and pleiotropy. The results suggest that interleukin-1 receptor antagonist (*IL-1RA*) and β-nerve growth factor (*B-NGF*) are risk factors for thyroid cancer, while macrophage colony-stimulating factor (*M-CSF*) has a protective effect. *IL-1RA*, *B-NGF*, and *M-CSF* play a key role in regulating the tumour microenvironment compared with 38 other inflammatory factors that do not show a clear correlation. *IL-1RA* may promote cancer cell proliferation by activating pro-inflammatory signalling pathways, while *B-NGF* may enhance angiogenesis and immune escape, accelerating tumour progression. Conversely, *M-CSF* may reduce thyroid cancer risk by enhancing the anti-tumour immune response. Additionally, single-nucleotide polymorphism survival prognostic analysis showed that specific genetic variants associated with *IL-1RA*, *B-NGF*, and *M-CSF* may influence overall survival (OS) and cancer-specific survival (CSS) in patients with thyroid cancer, further supporting their potential value in thyroid cancer management. These findings suggest that *IL-1RA* and *B-NGF* could serve as novel biomarkers for early detection and disease monitoring of thyroid cancer, while *M-CSF* could be a potential therapeutic target, providing theoretical support for personalised intervention strategies. The results provide a new direction for precision medicine research in thyroid cancer.

**Supplementary Information:**

The online version contains supplementary material available at 10.1007/s12672-025-03533-9.

## Introduction

The incidence of thyroid cancer, a significant global health issue, has been increasing in recent decades [1–3]. Although survival rates are generally high, treatment outcomes vary according to cancer subtype, stage at diagnosis, and patient age [4]. Current treatments, including surgery, radioactive iodine therapy, and thyroid hormone therapy, can cause hypothyroidism and lifelong medication dependence [5]. This highlights the need for a deeper understanding of the pathogenesis of thyroid cancer, identification of risk factors, and guidance for prevention and control, which are crucial for reducing the incidence and mortality rates of thyroid cancer and improving public health.

The aetiology of thyroid cancer is multifaceted and is believed to be associated with exposure to ionising radiation [6, 7], female reproductive factors [8], environmental pollution [9], dietary patterns [10], and lifestyle choices [11]. Moreover, inflammation is increasingly being recognised as a key factor in the development and metastasis of diverse cancers, including thyroid cancer [12]. Studies have shown that inflammatory cytokines and chemokines can modulate the tumour microenvironment, affecting tumour growth, angiogenesis, and immune evasion [13]. The overactivation of inflammatory markers, such as interleukins (IL), can contribute to the onset of thyroid cancer through systemic inflammation [14–16]. Previous clinical studies have suggested that IL-1β, IL-2, IL-27, and tumour necrosis factor (TNF)-α could serve as biomarkers for the diagnosis and monitoring of thyroid cancer progression [17]. Additionally, results from the European Prospective Investigation into Cancer and Nutrition (EPIC) indicate that IL-10, IL-12A, and IL-4I1 may serve as potential indicators for the onset of thyroid cancer [18–20]. IL-17 and TNF-α are potential prognostic markers for patients with Hashimoto's disease complicated by thyroid cancer [21]. Furthermore, vascular endothelial growth factor (VEGF) stimulates angiogenesis and increases vascular permeability, which is generally considered a marker of tumour invasiveness. Moreover, studies have found that increased *VEGF* expression in papillary thyroid carcinoma is positively correlated with serum levels of thyroid-stimulating hormone [22], and its genotype may play a protective role in the development of differentiated thyroid cancer [23]. In particular, VEGF-C and its receptor VEGFR-3 have been demonstrated to play a role in promoting lymphangiogenesis and angiogenesis, which are crucial for thyroid cancer progression and metastasis [24]. However, bidirectional Mendelian randomisation (MR) studies have not found a causal relationship between *VEGF* and thyroid cancer [25]. Although the role of VEGF in angiogenesis is well established, the lack of MR causality may reflect genetic heterogeneity in thyroid cancer subtypes or compensatory pathways in the tumour microenvironment. This may be limited by insufficient GWAS sample size, making the effect of VEGF-associated genetic variants on thyroid cancer risk difficult to detect. In addition, VEGF mainly affects the stage of tumour progression, while MR studies mainly explore long-term genetic effects, and it is difficult to capture its short-term dynamic effects. Based on the above, existing evidence on the relationship between inflammatory markers and thyroid cancer is inconsistent, primarily observational, and susceptible to confounding and reverse causality, which may hinder accurate causal inference. Thus, further research is required to clarify these connections.

**Fig. 1:**
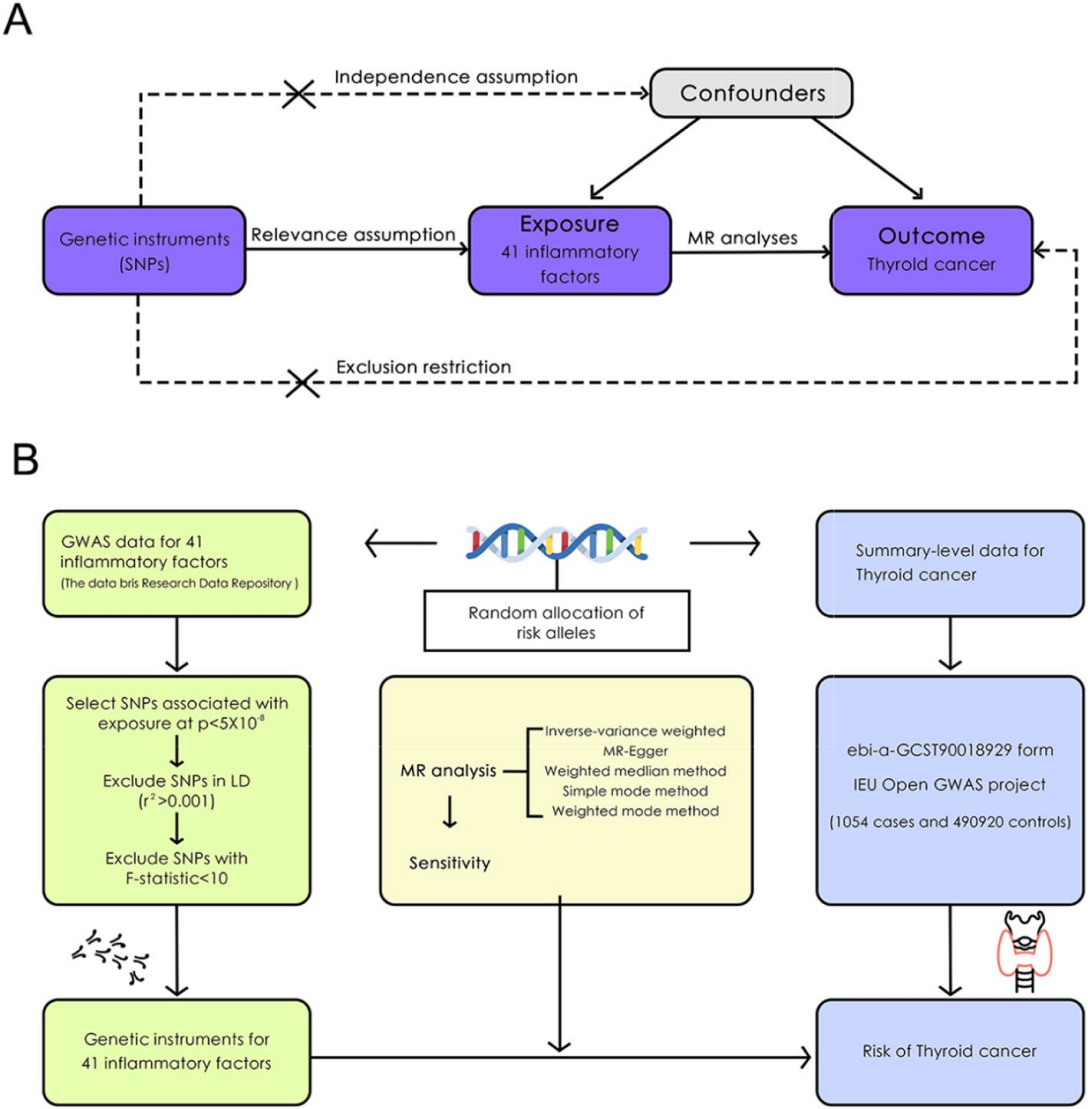
MR schematic illustrating the association between inflammatory factors and thyroid cancer. **a** The MR design is based on three key assumptions. **b** An overview of the analytical approach. MR, Mendelian randomisation

MR uses genetic variants as instrumental variables (IVs) to deduce causal effects and minimise reverse causality [26, 27]. With advancements in genome-wide association studies (GWAS) and molecular mechanisms, MR has been used extensively to explore disease risk factors [28, 29]. Currently, only one MR study has examined the link between inflammatory cytokines and autoimmune thyroid diseases, revealing that specific cytokine levels are linked to the risk of developing Graves’ disease and Hashimoto's thyroiditis [30]. However, the association between multiple inflammatory markers and thyroid cancer has not yet been explored. Therefore, this study aimed to investigate the causal relationship between 41 inflammatory factors and the risk of thyroid cancer using MR analysis, to identify potential key inflammatory factors and clarify their mechanisms in tumourigenesis. The study also aimed to provide new insights for the development of biomarkers and personalised treatment strategies. First, IL-1RA and B-NGF, as potential pathogenic factors for thyroid cancer, could be used for early risk prediction and as serum biomarkers for disease monitoring. Furthermore, the single-nucleotide polymorphisms (SNPs) associated with these inflammatory factors could be used to construct polygenic risk scores, thereby improving the early identification rate of high-risk populations. Second, macrophage colony-stimulating factor (M-CSF) may exert protective effects by regulating tumour-associated macrophages, suggesting its potential application in immunomodulatory therapy. Therefore, the study's findings could provide potential biomarkers for the early diagnosis of thyroid cancer and new therapeutic targets for precision treatment strategies, thereby enhancing the clinical value of personalised interventions. The study flow chart is shown in Fig. 1.

## Materials and methods

### MR study design

The study used MR analysis based on three basic assumptions: (1) Correlation: The selected genetic tool must have a significant and robust correlation with the exposure variable (inflammatory factor) to ensure that the instrumental variable can effectively predict exposure levels; (2) Independence: Genetic variation should be independent of any potential confounding factors, meaning that the association between instrumental variables and study results is established only by exposure variables and is not influenced by environmental factors or other genetic factors; (3) Exclusion restriction: Genetic variation should affect the outcome variable (thyroid cancer) only through exposure variables (inflammatory factors), and should not have indirect effects through other biological pathways, which may lead to horizontal pleiotropy and thus affect the accuracy of causal inference (Fig. 1a). These assumptions align with the MR principles described by Smith et al [31].

### Data sources

Relevant data were sourced from two publicly published GWAS databases (website accessed on 2023–11-03). The dataset on inflammatory markers was retrieved from the University of Bristol’s research data repository (https://data.bris.ac.uk/data/dataset/3g3i5smgghp0s2uvm1doflkx9x). This dataset contains a meta-analysis of summary statistics for the inflammatory cytokine GWAS performed in 8,293 participants from three Finnish cohorts (YFS: n = 2,136; FINRISK 1997: n = 3,021; FINRISK 2002: n = 3,136). Since the GWAS in ebi-a-GCST90018929 and the data.bris Research Data Repository are published studies, ethical committee approval and informed consent were not required for this study. All procedures were conducted in accordance with local ethical guidelines and regulations. In addition, the GWAS dataset is primarily based on thyroid cancer in European patients, the vast majority of whom have differentiated thyroid cancer, including papillary and follicular subtypes. Due to the small sample sizes of undifferentiated and medullary thyroid cancers in the GWAS data, the hypotheses and conclusions of this study mainly apply to differentiated thyroid cancer.

### Selection of IVs

Based on the three core assumptions, the selection of IVs was conducted as follows: meaningful SNPs were selected from the GWAS summary data of inflammatory factors, using P < 5 × 10^–8^ as the criterion for strong correlation; the linkage disequilibrium coefficient r^2^ was set at 0.001, and the linkage disequilibrium region width was defined as 10,000 kb to ensure the independence of SNPs and the pleiotropic effect on the results. SNPs associated with confounding factors and outcomes were excluded using Pheno Scanner; a threshold F-statistic value > 10 was used as the criterion to assess weak IVs. Relevant SNPs from the GWAS summary data on thyroid cancer were selected with a minimum r^2^ > 0.8. Information from these datasets was consolidated, excluding SNPs directly associated with thyroid cancer (P < 5 × 10^–8^). By following these IV selection criteria, the validity and reliability of the causal inference analysis were ensured (Fig. 1b).

### Statistical analysis

Statistical analysis was divided into three parts: Causal analysis, sensitivity analysis, and SNP prognostic evaluation. The primary analysis utilised five regression models: the random-effects IVW method, MR-Egger regression, WME, weighted model, and simple model, with SNPs as IVs, to verify the causal relationship between exposure (41 inflammatory markers) and outcome (thyroid cancer). The random-effects IVW method was primarily employed to analyse these causal relationships, with MR-Egger regression and the other three methods serving as supplementary analytical techniques. These methods were implemented using the TwoSampleMR 0.5.7 package in R software version 4.2.2, with a significance level of α = 0.05. The MR analysis code employed in this study is available at https://mrcieu.github.io/TwoSampleMR/articles/index.html.

Sensitivity analysis included heterogeneity and pleiotropy analyses. Cochran’s Q test was used to determine SNP heterogeneity; if P < 0.05, the results were considered heterogeneous. I^2^ (I-squared) is a statistic applied to measure heterogeneity, indicating the proportion of total variation attributed to heterogeneity. The I^2^ values ranged from 0 to 100%, with higher values reflecting greater heterogeneity. The formula for calculating I^2^ is I^2^ = (Q—Q_df)/Q. Pleiotropy analysis was assessed using the intercept from the MR-Egger method, where a non-zero intercept in the MR-Egger regression model (P > 0.05) indicated no evidence of pleiotropy. A leave-one-out analysis was performed by removing each SNP sequentially and reanalysing the remaining SNPs to assess the influence of each SNP on the results.

SNP prognosis survival analysis was conducted using the SUMMER online platform (http://njmu-edu.cn:3838/SUMMER/, Survival related cancer Multi-omics database via MEndelian Randomisation). The SUMMER system systematically evaluates the causal effects of risk factors and biomarkers on cancer survival, helping to elucidate the genetic mechanisms influencing cancer survival.

## Results

### Selection of SNPs as IVs

After a rigorous selection process, 836 SNPs were included in the thyroid cancer dataset. The F-statistics for individual SNPs ranged from 19.450 to 782.452 (mean: 32.364), suggesting a low likelihood that the causal association will be influenced by weak IVs.

### Causal relationship between 41 circulating inflammatory cytokines and thyroid cancer

Using the ebi-a-GCST90018929 dataset for MR analysis, the inverse variance weighted (IVW) analysis results indicated that IL-1 receptor antagonist (*IL-1RA*) and beta-nerve growth factor (*B-NGF*) were risk factors for thyroid cancer, whereas *M-CSF* acted as a protective factor. The remaining 38 inflammatory cytokines were not associated with the risk of thyroid cancer. Regarding the causal relationship between *IL-1RA* and thyroid cancer, the IVW model showed that for each unit increase in *IL-1RA*, the risk of developing thyroid cancer increased by 1.2164 times, with statistical significance (odds ratio [OR] = 1.2164, 95% confidence interval [CI]: 1.0020–1.4767, P = 0.0477). However, the MR-Egger regression, weighted median estimator (WME) analysis, weighted model, and simple model did not demonstrate any statistically significant results. Thus, based on the IVW results, *IL-1RA* was identified as a risk factor for thyroid carcinoma. For *B-NGF*, the IVW model indicated that each unit increase in *B-NGF* significantly raised the risk of thyroid cancer by 1.2436 times (OR = 1.2436, 95% CI: 1.0193–1.5173, P = 0.0317). Although the MR-Egger regression, WME analysis, weighted model, and simple model did not yield statistically significant differences, the IVW results consistently indicated that *B-NGF* was linked to an elevated risk of thyroid cancer.

**Fig. 2:**
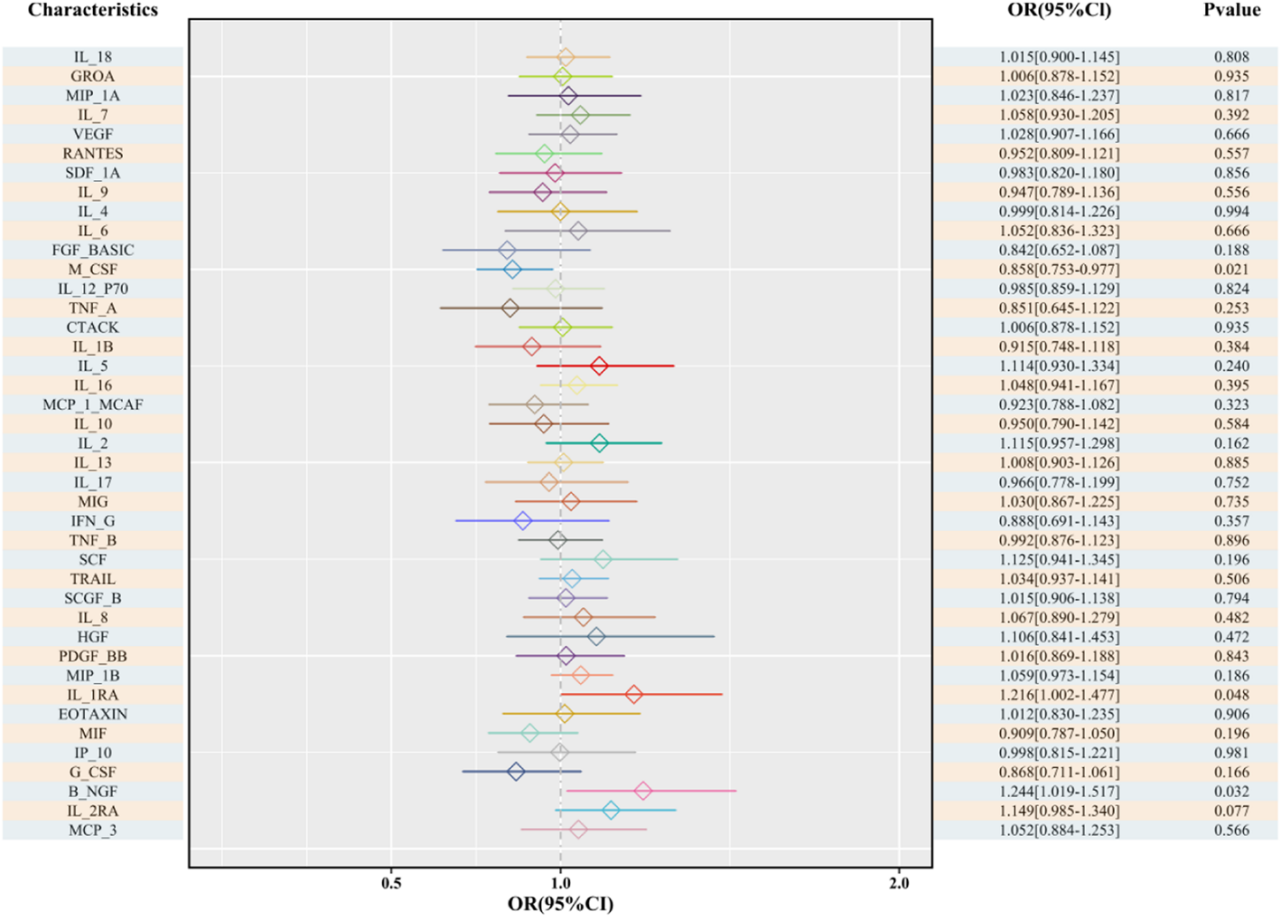
Forest plot of results from the MR regression analysis of causal associations. *B-NGF* beta nerve growth factor, *CTACK* cutaneous T cell-attracting chemokine, *FGFBasic* basic fibroblast growth factor, *GCSF* granulocyte colony-stimulating factor, *GROa* growth-regulated oncogene-a, *HGF* hepatocyte growth factor, *IFNg*, interferon-gamma, *IL* interleukin, *IF-10* interferon gamma-induced protein 10, *MCP1* monocyte chemotactic protein 1, *MCP3* monocyte-specific chemokine 3, *MCSF* macrophage colony-stimulating factor, *MIF* macrophage migration inhibitory factor, *MIG* monokine induced by interferon-gamma, *MIP1a* macrophage inflammatory protein-1a, *MIP1b* macrophage inflammatory protein-1b, *MR* Mendelian randomisation, *PDGFbb* platelet-derived growth factor-BB, *RANTES* regulated upon activation normal T cell expressed and secreted factor, *SCF* stem cell factor, *SCGFb* stem cell growth factor beta, *SDF1a* stromal cell-derived factor-1 alpha, *TNFa* tumour necrosis factor alpha, *TNFb* tumour necrosis factor beta, *TRAIL* TNF-related apoptosis-inducing ligand, *VEGF* vascular endothelial growth factor

During the causal analysis of *M-CSF* and thyroid cancer, the IVW model demonstrated that each unit increase in *M-CSF* significantly reduced the risk of developing thyroid cancer by 0.8577 times (OR = 0.8577, 95% CI 0.7531–0.9768, P = 0.0207). Notably, although the MR-Egger regression suggested a reduction in risk by 0.8053 times with each unit increase in *M-CSF*, this was not statistically significant (OR = 0.8053, 95% CI 0.5958–1.0885, P = 0.1809). Moreover, the WME analysis, weighted model, and simple model showed no statistically significant differences. Based on the IVW results, *M-CSF* was considered a protective factor against thyroid cancer. The remaining 38 inflammatory cytokines, including *IL-18*, did not correlate with the risk of thyroid cancer (Fig. 2). These results revealed an overall trend in the association between SNPs and thyroid carcinoma risk. To study the functional characteristics and biological correlations of the identified genes (*IL1RN*, *NGF*, and *CSF1*), we performed functional enrichment analysis, and the results are shown in Supplementary Figs. 1 and 2.

### Sensitivity analysis

**Fig. 3:**
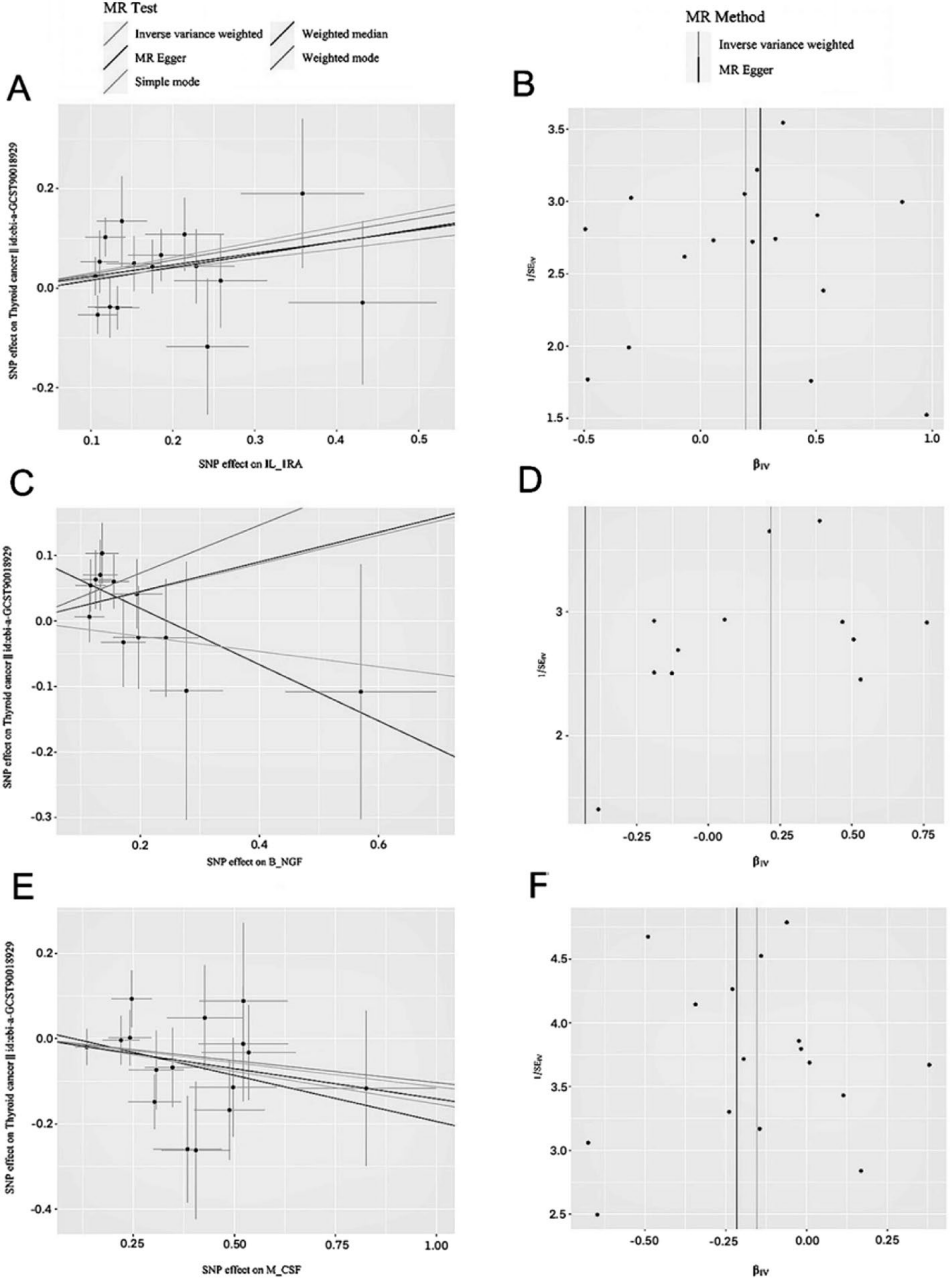
Scatter and funnel plots of MR analyses for *IL-1RA*, *B-NGF*, and *M-CSF* in thyroid cancer. **a**, **c**, **e** Scatter plots show individual instrumental variable (IV) associations with cytokine risk (black dots) plotted against their associations with thyroid cancer. The 95% confidence interval of the odds ratio for each IV is indicated by vertical and horizontal lines. The slope of the lines represents the estimated causal effect from the MR methods. **b**, **d**, **f** The funnel plots display the IV-weighted MR estimate of each cytokine single-nucleotide polymorphism with thyroid cancer versus 1/standard error (1/SEIV). MR, Mendelian randomisation; IV, inverse variance

Heterogeneity was evaluated using Cochran’s Q test and I^2^ statistic, with results indicating no significant heterogeneity in the *IL-1RA*, *B-NGF*, or *M-CSF* levels. The specific values are listed in Table 1. Pleiotropy was analysed using the intercepts from the MR-Egger regression model. The differences between the MR-Egger intercepts and those of *IL-1RA* and *B-NGF* were not statistically significant (Table 1). For *M-CSF*, the intercept difference from zero was also not statistically significant (Table 1). These results indicate no significant pleiotropy for the analysed factors. Scatter and funnel plots for *IL-1RA*, *B-NGF*, and *M-CSF* demonstrated that the SNP distributions were generally symmetrical, suggesting a low likelihood of bias impacting the causal associations (Fig. 3).

**Table 1:** MR heterogeneity and horizontal pleiotropy analysis results

Exposure	Heterogeneity			Horizontal pleiotropy		
	Cochran’s Q	P-value	I2	Egger intercept	SE	P-value
IL_ 18	25.8824	0.3592	7	− 0.0554	0.0229	0.0241
GROA	18.4992	0.4233	3	0.0004	0.029	0.9899
MIP_ 1A	14.5594	0.4836	0	0.0177	0.0397	0.6623
IL_7	8.2023	0.9755	0	− 0.0213	0.0303	0.4921
*VEGF*	42.477	0.0508	32	− 0.043	0.0214	0.0541
RANTES	14.0311	0.4474	0	0.0227	0.0395	0.5752
SDF_ 1A	16.1905	0.6445	0	− 0.0083	0.0205	0.692
IL_9	11.2793	0.664	0	0.0376	0.05	0.4649
IL_4	16.5025	0.7902	0	− 0.0327	0.0232	0.1732
IL_6	8.6216	0.9514	0	− 0.0111	0.0276	0.6935
FGF_BASIC	22.5791	0.2073	20	− 0.0161	0.0341	0.643
M_CSF	13.9998	0.5255	0	0.022	0.0485	0.6564
IL_ 12_P70	20.9747	0.694	0	− 0.0172	0.0177	0.3419
*TNF*_A	4.0829	0.3949	2	− 0.0737	0.0473	0.2169
CTACK	18.4992	0.4233	3	0.0004	0.029	0.9899
IL_ 1B	11.6061	0.2364	22	− 0.0453	0.0381	0.268
IL_5	25.7442	0.0792	34	0.0689	0.0378	0.0874
IL_ 16	16.0515	0.5202	0	0.0375	0.0255	0.1609
MCP_ 1_MCAF	20.4833	0.8462	0	− 0.0105	0.0244	0.671
IL_ 10	24.8774	0.2525	16	− 0.0341	0.0239	0.1688
IL_2	16.6731	0.612	0	− 0.0314	0.0273	0.2657
IL_ 13	18.6733	0.7692	0	− 0.0469	0.023	0.0526
IL_ 17	20.1966	0.3828	6	0.0349	0.0341	0.3189
MIG	21.2618	0.2664	15	− 0.0441	0.0365	0.2435
IFN_G	17.9669	0.3258	11	0.018	0.0317	0.5788
*TNF*_B	9.3287	0.5012	0	0.0284	0.0314	0.3896
SCF	17.0825	0.6476	0	− 0.0091	0.021	0.6699
TRAIL	25.4848	0.9532	0	− 0.0013	0.0145	0.9294
SCGF_B	23.5322	0.6561	0	− 0.0015	0.0234	0.9488
IL_8	7.951	0.7177	0	0.0196	0.0317	0.5504
HGF	4.0371	0.9457	0	0.0004	0.0447	0.9925
PDGF_BB	18.0934	0.8384	0	0.0192	0.0221	0.3921
MIP_ 1B	64.8503	0.3776	4	− 0.0042	0.0145	0.7725
IL_ 1RA	16.7561	0.3337	10	− 0.0103	0.047	0.8293
EOTAXIN	43.6546	0.0396	34	− 0.0263	0.0295	0.3802
MIF	21.1201	0.3302	10	− 0.0142	0.0254	0.5838
IP_ 10	17.1842	0.2465	19	− 0.0479	0.0392	0.2436
G_CSF	18.2313	0.507	0	0.0168	0.0206	0.4235
B_NGF	9.5794	0.5686	0	0.1052	0.0507	0.0648
IL_2RA	25.3342	0.1498	25	− 0.006	0.0288	0.8376
MCP_3	13.7179	0.0564	49	0.0163	0.0688	0.8201

**Fig. 4:**
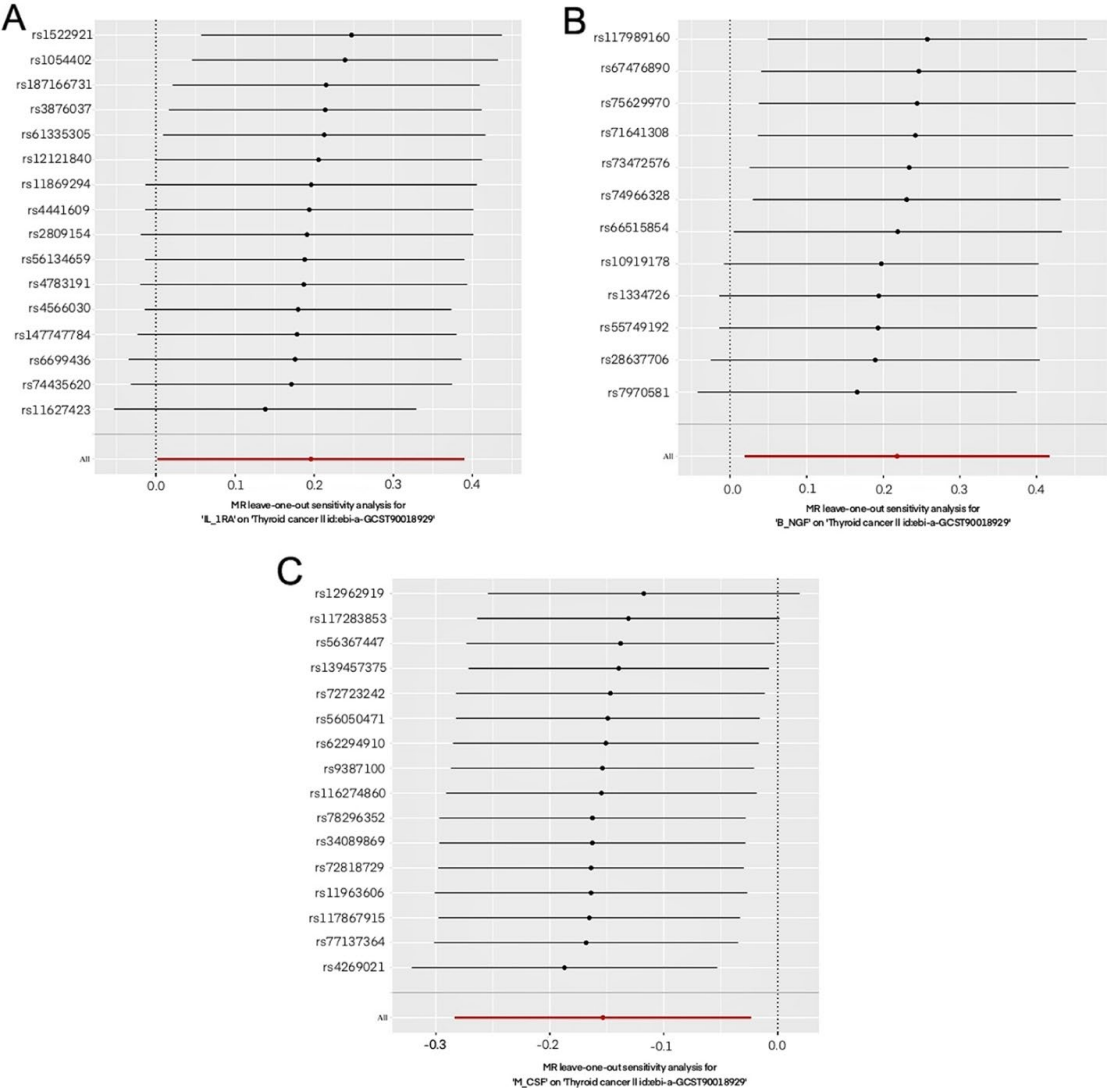
Results of the “leave-one-out” sensitivity analysis. **a** MR leave-one-out sensitivity analysis for *IL-1RA* in thyroid cancer. **b** MR leave-one-out sensitivity analysis for *B-NGF* in thyroid cancer. **c** MR leave-one-out sensitivity analysis for *M-CSF* in thyroid cancer. MR, Mendelian randomisation; *IL-1RA*, interleukin-1 receptor antagonist; *B-NGF*, beta-nerve growth factor; *M-CSF*, macrophage-colony stimulating factor

Sensitivity was assessed using the leave-one-out method, which involved sequentially excluding SNPs for *IL-1RA*, *B-NGF*, and *M-CSF* (Fig. 4). Our analysis results were consistent with those obtained when all SNPs were included, indicating that no single SNP had a meaningful effect on the causal estimates. This confirms the robustness of the MR results. In addition, the analyses of heterogeneity and pleiotropy performed on *IL-1RA*, *B-NGF*, and *M-CSF* were not statistically significant, suggesting that the results are reliable. Additionally, a sensitivity analysis further confirmed the stability of the findings.

### SNP prognosis analysis

SNP information was obtained from SNPs that were significantly associated with inflammatory factors related to thyroid cancer. SUMMER results identified rs71641308 as a risk factor for overall survival (OS) in patients with thyroid cancer (HR = 5.2395, P < 0.01); however, the results for cancer-specific survival (CSS) indicated that rs71641308 was not related to thyroid cancer prognosis (HR = 115,973.3083, P = 0.0977). The SNPs rs11869294, rs116274860, and rs62294910 were not associated with OS prognosis in thyroid cancer (HR = 1.8224, 10.1573, and 2.3126, respectively; P > 0.05); however, the CSS results suggested that rs11869294, rs116274860, and rs62294910 were risk factors for thyroid cancer prognosis (HR = 61.4871, 90.4400, and 304.5627, respectively; P < 0.05). In addition, the SNP rs187166731 was identified as a risk factor for OS (HR = 43.1384, P < 0.01) and CSS (HR = 23,470.9553, P < 0.05) in thyroid cancer (Table 2). These results suggest that *IL-1RA*, *B-NGF*, and *M-CSF* are relevant to the prognosis of thyroid cancer.

**Table 2:** SNP prognostic analysis results

	CHR	SNP	BP hg19	A1	A2	MAF	HR OS	SE OS	P value OS	HR CSS	SE CSS	P value CSS
*B-NGF*	1	rs71641308	78,086,718	T	C	0.0669	5.2395	0.516	0.0013	115,973.3083	7.0405	0.0977
	1	rs10919178	169,473,503	C	T	0.2893	1.1788	0.4152	0.692	1.499	0.8027	0.614
	1	rs74966328	235,707,209	A	G	0.0205	2.0023	1.0004	0.4877	4.5779	1.8037	0.399
	2	rs55749192	106,354,163	G	A	0.2283	1.2522	0.3723	0.5458	0.2682	1.0235	0.1984
	3	rs66515854	3,891,306	C	T	0.1341	0.8558	0.6158	0.8003	1.542	1.3104	0.741
	6	rs1334726	63,193,678	T	G	0.4045	1.2271	0.3952	0.6045	1.3071	0.8312	0.7473
	10	rs75629970	17,595,441	A	G	0.0335	2.1502	0.855	0.3706	3.9101	1.3621	0.3168
	15	rs67476890	62,791,494	T	C	0.0922	1.6044	0.6542	0.4699	0.4416	1.3291	0.5386
	19	rs28637706	34,285,368	T	G	0.3722	0.5674	0.4499	0.2077	1.2365	0.8405	0.8006
*IL-1RA*	1	rs6699436	62,803,032	A	G	0.1264	1.0279	0.6185	0.9645	0	10,210.7594	0.9983
	1	rs12121840	165,541,642	T	C	0.0523	1.8392	1.0578	0.5646	32.7178	2.5972	0.1793
	3	rs4441609	36,892,717	T	C	0.3911	0.452	0.4632	0.0865	0.5564	0.7554	0.4377
	7	rs1522921	29,149,995	C	G	0.4826	0.631	0.3924	0.2406	1.2754	0.6926	0.7254
	9	rs1054402	119,163,509	T	C	0.25	0.7587	0.5035	0.5834	0.0286	2.0492	0.0829
	11	rs74435620	108,436,318	G	A	0.1152	0.7881	0.6295	0.7052	0.4818	1.4691	0.6192
	13	rs2809154	84,727,524	T	C	0.1788	1.0951	0.5689	0.8731	1.9649	0.9622	0.4827
	13	rs4566030	114,907,255	G	A	0.1508	2.1585	0.5861	0.1893	2.0024	1.0193	0.4958
	14	rs11627423	33,200,623	C	A	0.3616	0.6572	0.4531	0.3541	0.0373	1.6946	0.0524
	15	rs61335305	66,453,074	A	C	0.0196	2.1545	1.1614	0.5087	9.1856	1.4149	0.117
	16	rs4783191	85,419,810	T	C	0.2151	0.7346	0.5255	0.5573	0.2421	1.2246	0.2468
	17	rs11869294	43,857,223	G	C	0.0698	1.8224	0.5479	0.2733	61.4871	1.7228	0.0168
	22	rs187166731	42,666,026	T	C	0.0115	43.1384	1.4554	0.0097	23,470.9	5.0814	0.0477
*M-CSF*	1	rs78296352	22,821,844	T	G	0.0391	2.6414	0.79	0.2189	0	10,849.1559	0.9986
	2	rs34089869	67,923,478	T	C	0.1006	0.5035	0.8578	0.4237	0	11,676.4 031	0.9987
	2	rs72818729	75,870,247	C	T	0.0961	0.4481	0.7913	0.3103	1.4011	1.355	0.8034
	3	rs77137364	74,989,832	G	C	0.0419	3.0283	0.9021	0.2194	5.4908	1.4377	0.2362
	3	rs116274860	148,392,817	G	T	0.0197	10.1573	1.275	0.069	90.44	2.1018	0.0321
	3	rs62294910	182,198,339	A	G	0.0531	2.3126	0.9238	0.3641	304.5627	2.4825	0.0212
	5	rs72723242	7,262,954	T	G	0.0307	1.1369	1.1388	0.9103	0	13,290.0 502	0.9988
	6	rs11963606	4,592,689	G	C	0.0141	0	8163.3 586	0.9983	0	12,773.5 364	0.9989
	6	rs9387100	113,102,954	T	C	0.3408	0.3584	0.5599	0.0668	0.2234	1.0059	0.1362
	8	rs56367447	3,871,527	T	C	0.0426	1.672	0.7852	0.5127	1.2679	1.4293	0.8681
	12	rs117283853	30,965,225	G	A	0.0343	1.29	0.896	0.7762	0	15,968.7 705	0.9991
	18	rs12962919	75,778,756	T	C	0.0971	1.6449	0.6955	0.4743	0	14,037.2 837	0.9989
	22	rs4269021	47,673,948	G	C	0.1376	0.1557	1.1495	0.1057	0.5525	1.4633	0.6851

*CHR* chromosome, *SNP* single nucleotide polymorphism, *BP_hg19* position of the SNP on the hg19 reference genome, *A1* effect allele, *A2* other allele, *MAF* minor allele frequency, *HR_OS* hazard ratio for overall survival, *SE_OS* standard error for overall survival analysis, *P_value_OS* p-value for overall survival analysis, *HR_CSS* hazard ratio for cancer-specific survival, *SE_CSS* standard error for cancer-specific survival analysis, *P-value_CSS* p-value for cancer-specific survival analysis

## Discussion

As a disease affecting the endocrine system, thyroid cancer can lead to various health complications, including hormonal imbalances. If left untreated, it may metastasise to other parts of the body, causing further damage [32]. Additionally, inflammation is a key process in the pathogenesis of various cancers, including thyroid cancer [33]. Therefore, exploring the causal relationship between inflammatory factors and thyroid cancer may provide new directions for developing novel diagnostic and therapeutic strategies. In this study, we identified 836 SNPs as IVs using MR to explore the causal relationship between 41 circulating inflammatory factors and thyroid cancer risk. These results indicated that *IL-1RA* and *B-NGF* are risk factors for the onset of thyroid cancer, whereas *M-CSF* serves as a protective factor. To date, our research is the first to identify and establish a causal relationship between circulating inflammatory factors and thyroid cancer.

IL-1RA regulates the inflammatory response mainly by competitively inhibiting the receptor binding of IL-1α and IL-1β and affecting signalling pathways such as NF-κB [34]. IL-1RA plays different roles in various types of cancer [35]. For example, in oral squamous cell carcinoma, it promotes tumour progression by affecting mitochondrial metabolism and is associated with a poor prognosis, potentially influencing cancer cell energy supply and proliferation [36]. In colorectal cancer, IL-1RA reduces cancer cell metastatic potential by inhibiting the CXCL12/CXCR4 signalling axis, suggesting that it may have anti-metastatic effects in certain cancers [37]. In thyroid cancer, particularly in anaplastic thyroid carcinoma and follicular thyroid carcinoma, elevated IL-1RA may influence the tumour microenvironment through the IL-1β signalling pathway, altering inflammatory responses and immune cell infiltration, thereby regulating tumour cell growth and differentiation [38]. This indicates that the role of IL-1RA in thyroid cancer may be more complex, with different inflammatory states and immune regulation determining its specific effects [39, 40]. The duality of IL-1RA in tumour biology suggests that although IL-1RA can prevent tumour progression by inhibiting the inflammatory response in some cases, in others, it instead promotes tumour growth and metastasis by suppressing the anti-tumour response of the immune system. The specific manifestations of this duality in thyroid cancer have not been fully elucidated; however, IL-1RA has been proposed to influence cancer progression through a complex cytokine network and immune escape mechanisms [41].

In summary, the role of IL-1RA in thyroid cancer is similar to that of other cancers, although it also has its own unique pathway and mechanism, which provides a theoretical basis for its use as a potential therapeutic target. Future studies could further explore the specific molecular mechanisms of IL-1RA in thyroid cancer to help better understand its role in cancer progression and develop new therapeutic strategies.

*B-NGF* primarily promotes the growth, differentiation, and maturation of central and peripheral neurons, all while maintaining the physiological functions of the nervous system [42, 43]. Moreover, recent studies have shown that it also plays an important role in various non-neurological physiological and pathological processes, including tumourigenesis. B-NGF promotes tumour progression by enhancing angiogenesis and cell survival [44]. Additionally, B-NGF is involved in immunomodulation, directly affecting innate and adaptive immune responses in B and T cells, as well as regulating immune system activation in tissues [45]. These findings suggest that B-NGF likely plays a role in the pathogenesis of thyroid cancer by promoting the proliferation and differentiation of tumour cells, as well as regulating immune responses. This is consistent with our finding that *B-NGF* was associated with an increased risk of thyroid cancer. Thus, we highlight the importance of *B-NGF* as a risk factor for thyroid cancer and suggest that it may serve as a novel target for cancer therapy.

In contrast, in our study, a reduced risk of thyroid cancer was associated with *M-CSF*, which influences macrophage differentiation and survival. Typically, *M-CSF* is commonly overexpressed in tumours and is thought to enhance tumour growth and invasiveness by stimulating the growth and differentiation of tumour-associated macrophages [46]. Interestingly, our findings identifying *M-CSF* as a protective factor against thyroid cancer are consistent with previous studies on the complex role of *M-CSF* in carcinogenesis. M-CSF’s protective effect might arise from its ability to polarise tumour-associated macrophages (TAMs) toward an anti-tumour M1 phenotype in thyroid microenvironments, as shown in colorectal cancer models [47]. IL-12 secreted during polarisation stimulates cytotoxic T cells and counteracts IL-1RA-mediated immunosuppression, suggesting a therapeutic synergy between M-CSF agonists and IL-1RA inhibitors [48]. Under certain conditions, M-CSF may inhibit tumour growth and metastasis, possibly by modulating inflammatory responses and promoting immune surveillance to reduce the risk of thyroid cancer [49, 50]. Thus, the observed protective effects of *M-CSF* may facilitate the development of immunomodulatory therapies for thyroid cancer. Overall, the dual role of inflammation in cancer has been further recognised, serving as a driver of tumour progression and a mediator of immune surveillance.

In addition, IL-1RA, B-NGF, and M-CSF may interact in the progression of thyroid cancer to form a complex inflammatory network. For example, IL-1RA promotes inflammation through the NF-κB axis and may activate B-NGF expression, which enhances cell survival and angiogenesis [51]. In addition, B-NGF activates the PI3K/AKT/mTOR signalling pathway mainly through TrkA receptors, further promoting cancer cell proliferation and immune escape. On the other hand, M-CSF can regulate the tumour microenvironment through the CSF-1R receptor and affect the polarisation state of macrophages. In thyroid cancer, M-CSF may promote M1-type macrophage activation, thereby reducing IL-1RA-induced immunosuppression and forming a countervailing mechanism [52]. Therefore, IL-1RA, B-NGF, and M-CSF may form a dynamic balance in the pathological process of thyroid cancer.

Other inflammatory markers involved in this study, such as *IL-18*, were not found to have a causal relationship with the development of thyroid cancer, suggesting that thyroid cancer may be influenced by specific inflammatory pathways rather than generalised inflammatory activity. Moreover, other cytokines, although involved in inflammation, may not have a direct impact on thyroid cancer development, or their role is masked by compensatory mechanisms in the tumour microenvironment. Their potential role in thyroid cancer will be explored in future studies.

Our study also revealed a significant role of specific SNPs in the prognosis of thyroid cancer. For instance, SNP rs71641308, located in *B-NGF*, was found to be associated with OS in thyroid cancer, whereas SNP rs11869294, located in *IL-1RA*, and SNPs rs116274860 and rs62294910, located in *M-CSF*, were associated with CSS. These factors affecting OS and CSS provide valuable insights into the genetic landscape of thyroid cancer and highlight the potential of personalised medical approaches to improve patient management and treatment strategies. Our findings suggest that individuals with genetic predispositions (e.g., rs11869294 in IL-1RA or rs71641308 in B-NGF) could benefit from enhanced surveillance, such as regular thyroid ultrasounds or serum cytokine profiling. For populations with familial thyroid cancer, integrating these SNPs into polygenic risk scores may refine early detection strategies. Furthermore, targeting IL-1RA or B-NGF with monoclonal antibodies (e.g., anakinra for IL-1RA inhibition) could synergise with existing therapies like tyrosine kinase inhibitors, potentially overcoming drug resistance in advanced cases.

The validity of our findings was strengthened by the use of a large European cohort and SNPs, which are closely associated with inflammatory markers. Furthermore, the absence of significant heterogeneity or pleiotropy confirms the robustness of these association results. In summary, our findings offer important evidence for the causal relationship between inflammatory factors and the risk of developing thyroid cancer, not only confirming the critical role of inflammation in thyroid cancer but also providing a genetic framework for understanding individual differences in disease prognosis. These findings may help optimise screening strategies for people at high risk of thyroid cancer. For example, IL-1RA and B-NGF can be used as genetic risk markers to improve disease prediction by combining traditional thyroid cancer screening with polygenic risk score methods. In addition, M-CSF may be combined with existing immunotherapy strategies as part of targeted macrophage functional regulation to adjust the balance of inflammation in the tumour microenvironment. These findings provide new ideas for personalised intervention of thyroid cancer and deserve further research.

However, this study has some limitations. First, this study was based on MR analyses of GWAS data from a European population, limiting generalisability to other ethnicities. Genetic variations in inflammatory pathways and environmental factors may modulate cytokine-cancer associations. Future trans-ethnic MR studies are needed to validate these findings. Second, although sensitivity analyses suggested minimal pleiotropy, residual bias from horizontal pleiotropy cannot be fully excluded. For instance, SNPs in IL-1RA may influence thyroid cancer through pathways unrelated to inflammation (e.g., metabolic regulation). Future studies using multivariable MR or colocalisation analysis could further disentangle these effects. Third, due to the complex aetiology of thyroid cancer, circulating inflammatory factors cannot fully explain its pathogenesis, and comprehensive data are needed to better guide its clinical treatment. These limitations highlight the need for further studies to validate and expand our findings.

This study confirmed that *IL-1RA* and *B-NGF* are risk factors for thyroid cancer, whereas *M-CSF* is a protective factor. These findings lay a foundation for future research, suggesting the potential use of inflammatory factors as biomarkers or therapeutic targets. Clinical trials testing IL-1RA inhibitors or M-CSF-based therapies could translate these findings into improved patient outcomes and new directions for developing more personalised therapeutic strategies.

## Supplementary Information

Supplementary material 1.

## Data Availability

The datasets used in this study are derived from publicly available data sources. These data can be accessed via the following URLs/platforms: https://data.bris.ac.uk/data/dataset/3g3i5smgghp0s2uvm1doflkx9x and https://gwas.mrcieu.ac.uk/.
