# Supplementary material for: Mendelian randomization analysis reveals causal roles of inflammatory cytokines in thyroid cancer pathogenesis
Source: Discov Oncol. 2025 Sep 2;16:1671. doi: 10.1007/s12672-025-03533-9 (PMC12405125; doi:10.1007/s12672-025-03533-9)
Supplement: Supplementary file 1 — Supplementary material 1. [file 12672_2025_3533_MOESM1_ESM.docx]

**Inflammatory factors in the pathogenesis of thyroid cancer: A Mendelian randomisation study**

**Short title: Pathogenesis of thyroid cancer**

Bo Liu,^1†^ Tingting Zhang,^2†^ Jihua Han,^1^ Wen Bi,^1^ Chunlei Nie,^1^ and Jiewu Zhang^1*^

^1^Department of Head and Neck Surgery, Harbin Medical University Cancer Hospital，No.150 Haping Road, Nangang District, Harbin, 150081, China

^2^Psychology and Health Management Centre, Harbin Medical University, No.157, Baojian Road, Nangang District, Harbin, 150076, China

***Corresponding author**

Jiewu Zhang

Department of Head and Neck Surgery, Harbin Medical University Cancer Hospital，No.150 Haping Road, Nangang District, Harbin, 150081, China

Email: [zhang_jwu_nic@126.com](mailto:zhang_jwu_nic@126.com) (JZ)

**Supplementary Figure**


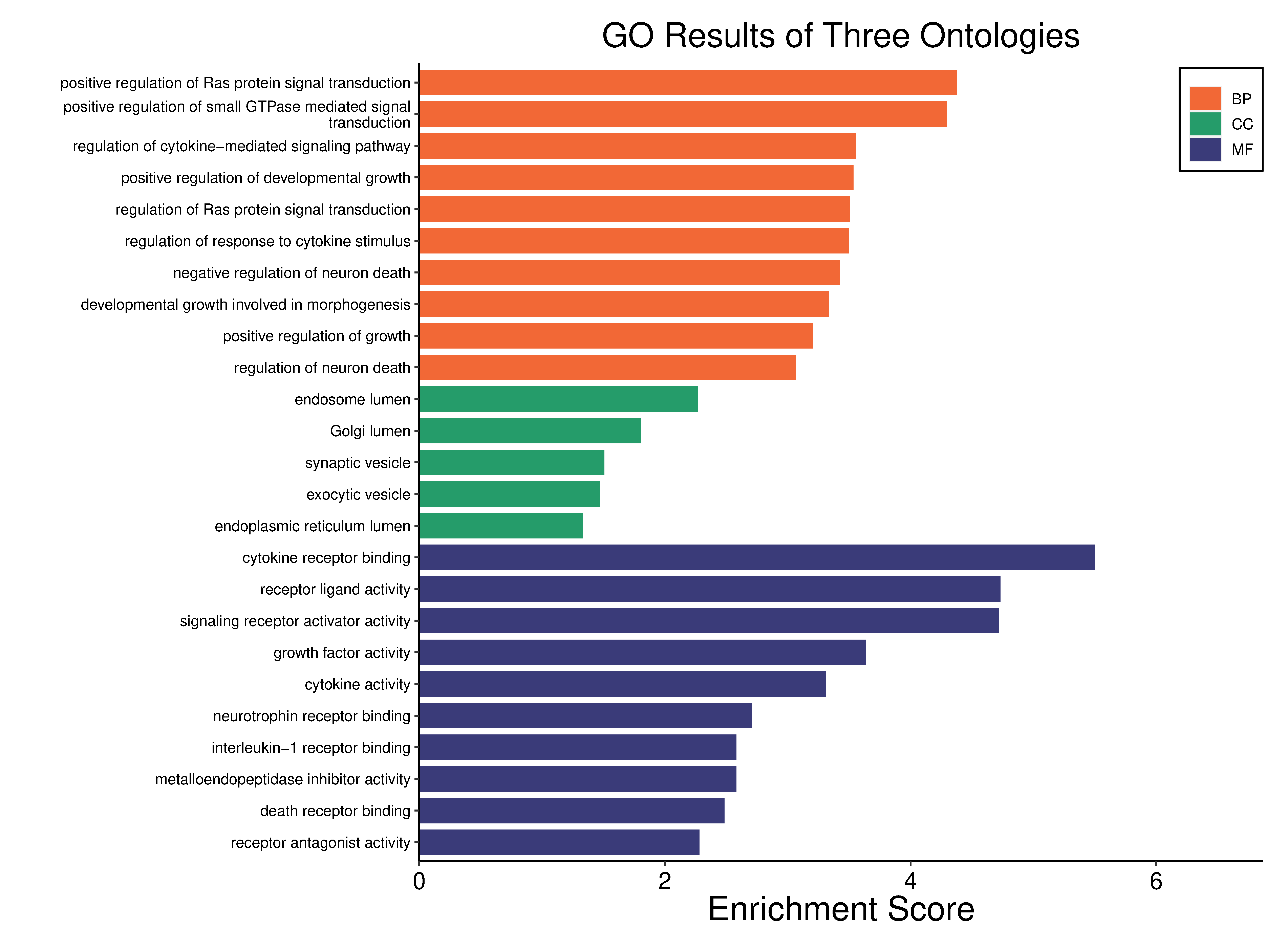


**Supplementary Fig. 1.** GO Enrichment Analysis of IL1RN, NGF, and CSF1 in Biological Processes, Cellular Components, and Molecular Functions.


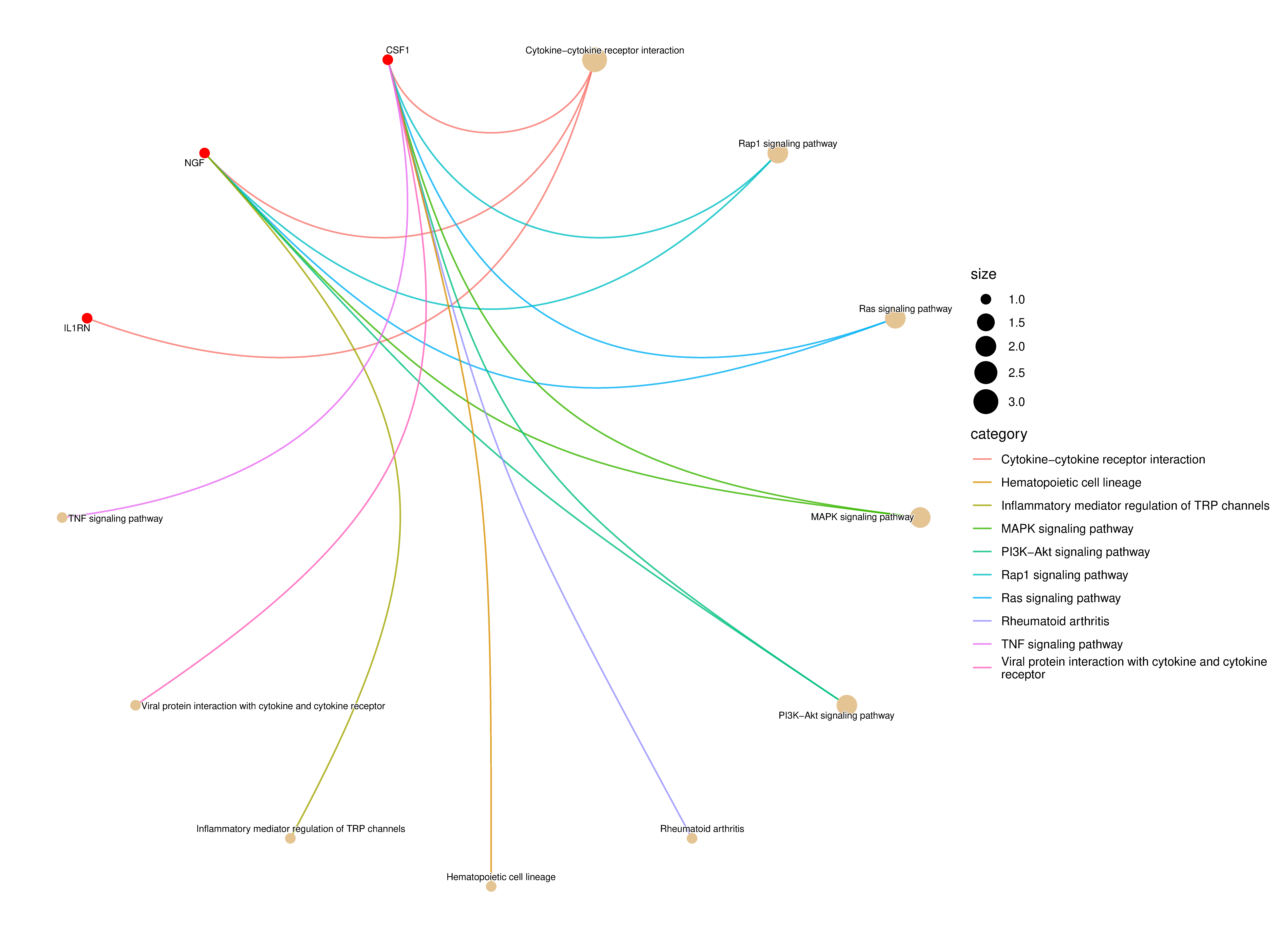


**Supplementary Fig. 2.** KEGG Pathway Enrichment Analysis of IL1RN, NGF, and CSF1: Gene-Pathway Interaction Network.
